# Control of Fluoroquinolone Resistance through Successful Regulation, Australia

**DOI:** 10.3201/eid1809.111515

**Published:** 2012-09

**Authors:** Allen C. Cheng, John Turnidge, Peter Collignon, David Looke, Mary Barton, Thomas Gottlieb

**Affiliations:** Monash University, Melbourne, Victoria, Australia (A.C. Cheng);; Alfred Hospital, Melbourne (A.C. Cheng);; Women’s and Children’s Hospital, Adelaide, South Australia, Australia (J. Turnidge);; University of Adelaide, Adelaide (J. Turnidge);; The Canberra Hospital, Garran, Canberra, Australia (P. Collignon);; Australian National University, Canberra (P. Collignon);; Princess Alexandra Hospital, Brisbane, Queensland, Australia (D. Looke);; University of Queensland, Brisbane (D. Looke);; University of South Australia, Adelaide (M. Barton);; Concord Hospital, Sydney, New South Wales, Australia (T. Gottlieb);; and University of Sydney, Sydney (T. Gottlieb)

**Keywords:** bacterial drug resistance, antimicrobial drug resistance, fluoroquinolones, health policy, human, animal, bacteria, Australia

## Abstract

Restricted Fluoroquinolone use in humans and food animals has result in low rates of resistance in human pathogens

## Medscape CME Activity

Medscape, LLC is pleased to provide online continuing medical education (CME) for this journal article, allowing clinicians the opportunity to earn CME credit.

This activity has been planned and implemented in accordance with the Essential Areas and policies of the Accreditation Council for Continuing Medical Education through the joint sponsorship of Medscape, LLC and Emerging Infectious Diseases. Medscape, LLC is accredited by the ACCME to provide continuing medical education for physicians.

Medscape, LLC designates this Journal-based CME activity for a maximum of 1 *AMA PRA Category 1 Credit(s)*^TM^. Physicians should claim only the credit commensurate with the extent of their participation in the activity.

All other clinicians completing this activity will be issued a certificate of participation. To participate in this journal CME activity: (1) review the learning objectives and author disclosures; (2) study the education content; (3) take the post-test with a 70% minimum passing score and complete the evaluation at www.medscape.org/journal/eid; (4) view/print certificate.

**Release date: August 16, 2012; Expiration date: August 16, 2013**

## Learning Objectives

Upon completion of this activity, participants will be able to:

- Describe restrictions in Australia regarding use of fluoroquinolones, based on a review
- Describe development of fluoroquinolone resistance in Australia compared to that in other countries, based on a review
- Describe potential harms of restricting fluoroquinolone use, and strategies to eliminate those harms, based on a review

## CME Editor

**Jean Michaels Jones,** Technical Writer/Editor, *Emerging Infectious Diseases. Disclosure: Jean Michaels Jones has disclosed no relevant financial relationships.*

## CME Author

**Laurie Barclay, MD,** freelance writer and reviewer, Medscape, LLC. *Disclosure: Laurie Barclay, MD, has disclosed no relevant financial relationships.*

## Authors

*Disclosures:* ***Allen C. Cheng, PhD; Peter Collignon, FRACP; David Looke, FRACP;*** *and* ***Mary Barton, PhD,*** *have disclosed no relevant financial relationships.* ***John Turnidge, FRACP,*** *has disclosed the following relevant financial relationships: served as an advisor or consultant for AstraZeneca Australia, BioMerieux.* ***Thomas Gottlieb, FRACP,*** *has disclosed the following relevant financial relationships: served as an advisor or consultant for AstraZeneca Australia, Janssen-Cilag, Novartis, Pfizer.*
