# Supplementary material for: Control of Fluoroquinolone Resistance through Successful Regulation, Australia
Source: Emerg Infect Dis. 2012 Sep;18(9):1453–60. doi: 10.3201/eid1809.111515 (PMC3437704; doi:10.3201/eid1809.111515)
Supplement: Related texts

# Review article: Control of Fluoroquinolone Resistance through Successful Regulation, Australia

**DOI:** 10.3201/eid1809.111515

**Authors:** Allen C. Cheng, John Turnidge, Peter Collignon, David Looke, Mary Barton, Thomas Gottlieb

**Affiliations:** Monash University, Melbourne, Victoria, Australia (A.C. Cheng);; Alfred Hospital, Melbourne (A.C. Cheng);; Women’s and Children’s Hospital, Adelaide, South Australia, Australia (J. Turnidge);; University of Adelaide, Adelaide (J. Turnidge);; The Canberra Hospital, Garran, Canberra, Australia (P. Collignon);; Australian National University, Canberra (P. Collignon);; Princess Alexandra Hospital, Brisbane, Queensland, Australia (D. Looke);; University of Queensland, Brisbane (D. Looke);; University of South Australia, Adelaide (M. Barton);; Concord Hospital, Sydney, New South Wales, Australia (T. Gottlieb);; and University of Sydney, Sydney (T. Gottlieb)

## Abstract

Fluoroquinolone antimicrobial drugs are highly bioavailable, broad-spectrum agents with activity against gram-negative pathogens, especially those resistant to other classes of antimicrobial drugs. Australia has restricted the use of quinolones in humans through its national pharmaceutical subsidy scheme; and, through regulation, has not permitted the use of quinolones in food-producing animals. As a consequence, resistance to fluoroquinolones in the community has been slow to emerge and has remained at low levels in key pathogens, such as *Escherichia coli*. In contrast to policies in most other countries, this policy has successfully preserved the utility of this class of antimicrobial drugs for treatment of most infections.

Nalidixic acid, the first quinolone introduced into clinical practice, was developed in the 1960s; its use was largely confined to the treatment of urinary tract infections. After the development of several fluoroquinolone antimicrobial drugs, including ciprofloxacin and norfloxacin in the 1980s, and then ofloxacin and levofloxacin, and more recently gatifloxacin and moxifloxacin, the use of this class of antimicrobial drugs increased greatly worldwide. Estimates from the late 1990s suggested that quinolones were the most prescribed antibacterial agent worldwide (*1*). Soon after these drugs were registered, the government of Australia developed policies to restrict use of quinolone antimicrobial drugs by humans and to prevent their use in food-producing animals. These policies have been associated with low rates of resistance to this valuable antimicrobial drug class in Australia.

## Usefulness of Fluoroquinolones

Quinolone antimicrobial drugs are commonly used as first-line empiric therapy for urinary tract infections, upper and lower respiratory tract infections, enteric infections, and gonococcal infection. They are particularly useful against deep infections caused by gram-negative bacteria, including those, such as *Pseudomonas* spp., that are resistant to other orally administered antimicrobial drugs. Specific quinolone antimicrobial drugs administered to pets and food-producing animals are known to transmit cross-resistance to humans (*1*).

## Contribution of Fluoroquinolone Use to Fluoroquinolone Resistance

Quinolones act by inhibiting bacterial DNA gyrase and/or topoisomerase IV (*2*). Target modification is a common mechanism for resistance, in which >1 point mutations in the *gyrA* or *parC* genes generate unequivocal resistance. This mutation can be induced in vitro by exposure to antimicrobial drug concentrations of >8× the MIC (*3*). Other mechanisms can also mediate resistance, including decreased expression of porins, leading to decreased membrane permeability, and overexpression of antimicrobial drug efflux pumps (*2*). The transfer of quinolone resistance by mobile genetic elements has the potential to rapidly disseminate resistance, and its contribution to the spread of resistance is being increasingly recognized (*4*). Under certain circumstances, resistance to fluoroquinolones can emerge during treatment. Some studies have reported that < 50% of all patients taking quinolones for prostatitis are colonized with quinolone-resistant *Escherichia coli* strains (*5*) and have described quinolone resistance after treatment courses of as few as 3 days (*6*). Whether resistance is caused by de novo resistance mutations or amplification of resistant strains already present in low numbers is not known. Furthermore, even parenteral fluoroquinolones are actively excreted into the intestine and may select for resistance in normal intestinal flora.

Although other factors are likely to contribute to resistance in persons, ecologic data show an association between fluoroquinolone use and resistance. This finding is supported by differences between the usual habitats of certain bacterial species and the effect fluoroquinolone use has on resistance development. Because some bacteria, such as *Streptococcus pneumoniae, Neisseria gonorrhoeae*, and *Salmonella enterica* serovar Typhi are transmitted from human to human, resistance in these organisms is likely to indicate human use of antimicrobial drugs and consequent antimicrobial drug selection pressure. Resistance in *N. gonorrhoeae* and *S.* Typhi are also influenced by variations in global epidemiology of disease and in ease of availability of quinolone antimicrobial drugs, including over-the-counter access in Asia, where much higher levels of resistance have been documented (*7*). Resistance in *Campylobacter* spp. and non-typhoidal salmonella is more likely to reflect antimicrobial drug administration to food-producing animals (*8*). *E. coli* is widely distributed among humans, animals, water, and some foods; thus, selection pressure is likely to be exerted by antimicrobial drug use in human and agricultural sectors. This likelihood is supported by molecular typing studies in which researchers examined *E. coli* strains resistant to trimethoprim-sulfamethoxazole, quinolones, and extended-spectrum cephalosporins in humans and in commercial poultry products in the United States, where these antimicrobial drugs are or have been used in poultry production (*9*). The authors found that resistant strains in humans were more closely aligned with resistant isolates in poultry than to susceptible human strains, suggesting that the resistant strains in humans were most likely to be of poultry origin.

## Low Use of Quinolone by Humans and Prohibition of Its Use in Food-producing Animals in Australia

Three quinolones are available for systemic use in humans in Australia: norfloxacin, ciprofloxacin, and moxifloxacin. Other quinolones have been available in the past (nalidixic acid, enoxacin, trovafloxacin, and gatifloxacin) but have been withdrawn from the market for a variety of reasons. In Australia, national guidelines for antimicrobial drug use in humans have been published and expanded since 1976. Indications for antimicrobial use are reviewed by a panel of infectious diseases experts approximately every 3 years (*10*). These guidelines are widely promulgated and generally accepted as a standard for prescribing antimicrobial drugs in the community and in hospitals.

The use of quinolone antimicrobial drugs in Australia has been actively constrained by guidelines that recognize their status as a reserve antimicrobial drug. For example, in the current guidelines, ciprofloxacin is not listed as an option in the management of lower urinary tract infection, and it is listed as a treatment for acute pyelonephritis only when resistance to all other recommended drugs is proven or the causative organism is *Pseudomonas aeruginosa*. For treatment of foot infections in persons with diabetes, ciprofloxacin is only recommended as an alternative for patients with penicillin hypersensitivity; the drug is listed for water-related infections caused by *Aeromonas* spp., but is not listed for treatment of wounds caused by other organisms. For respiratory infections, moxifloxacin is not listed as an option for the empiric treatment of community-acquired pneumonia in outpatients, except in patients who have severe penicillin hypersensitivity; ciprofloxacin is listed as an option to treat Legionella infection and in directed therapy for infections in which a susceptible pathogen has been identified. In almost all other countries, quinolones have been freely available and used for a broad range of indications as first-line therapy and have been promoted in treatment guidelines for conditions such as community-acquired pneumonia and uncomplicated urinary tract infections (*11*,*12*)

Australia has a subsidized outpatient pharmaceutical plan, the Pharmaceutical Benefits Scheme (PBS). Relatively expensive drugs (more than AU$30, in 2010 dollars) are not used widely unless prescribed by doctors according to indications listed by PBS. After 1988, ciprofloxacin use was subsidized by the PBS for “serious infections for which no other oral antimicrobial agent is appropriate.” In response to growing expenditures in the early 1990s, the Pharmaceutical Benefits Advisory Committee consulted with the National Health and Medical Research Council Working Party on Antibiotics, which suggested that specific indications would result in a more targeted use of quinolones. This suggestion was subsequently adopted by the PBS, and the list of indications underwent several modifications over the years, eventually leading to the PBS authority indications listed in Table 1.

**Table 1:** Quinolone antimicrobial drugs available for use in humans in Australia under the authority of the PBS*

Drug/route of administration	PBS listed indication	Consumer cost in AU$			Generic available
		Private market†	PBS GB	PBS CB	
Ciprofloxacin					
Oral	Respiratory tract infection proven or suspected to be caused by *Pseudomonas aeruginosa* in severely immunocompromised patients	25.20	34.20	5.60	Yes
	Bacterial gastroenteritis in severely immunocompromised patients				
	Infections proven to be caused by *P. aeruginosa* or other gram-negative bacteria resistant to all other oral antimicrobial drugs				
	Joint and bone infections, epididymo-orchitis, prostatitis, or perichondritis of the pinna suspected or proven to be caused by gram-negative or -positive bacteria resistant to all other appropriate antimicrobial drugs				
	Gonorrhea				
Topical, ear	Treatment of chronic suppurative otitis media in Aborigines or Torres Strait Islanders >1 mo of age	24.51	19.38	5.60	No
	Treatment of chronic suppurative otitis media in a patient <18 y of age with perforation of the tympanic membrane				
	Treatment of chronic suppurative otitis media in a patient <18 y of age with a grommet in situ				
Topical, eye	Bacterial keratitis	33.71	28.58	5.60	No
Ofloxacin					
Topical, eye	Bacterial keratitis	35.40	32.24	5.60	No
Norfloxacin					
Oral	Acute bacterial enterocolitis; complicated urinary tract infection	31.68	17.16	5.60	Yes
Moxifloxacin‡					
Oral	No longer listed	70.65	NA	NA	No

*PBS, Pharmaceutical Benefits Scheme; GB, general beneficiaries; CB, concessional beneficiaries, including pensioners; NA, no longer available in PBS. †Price to consumer varies between retail outlets. ‡Data from 2011.

The quinolones used in the treatment of respiratory infections, moxifloxacin and gatifloxacin, were approved for use in Australia in 2001. The Expert Advisory Group on Antimicrobial Resistance, the successor to the Working Party on Antibiotics, advised the Pharmaceutical Benefits Advisory Committee on the listing of moxifloxacin. For a few years, moxifloxacin was available only with authorization for treatment of *S. pneumoniae* pneumonia with proven penicillin resistance and was voluntarily withdrawn from the PBS by the manufacturer for marketing reasons. Moxifloxacin continues to be promoted by industry for use in hospitals and on the private (non-PBS) market, but estimated community use remains low (Figure 1). No applications for use in Australia have been made for other quinolones used widely elsewhere in the world, including oral ofloxacin and levofloxacin.

**Figure 1:** Data from Drug Utilization Sub-Committee Drug Utilization Database on Pharmaceutical Benefits Scheme and the Repatriation Pharmaceutical Benefits Scheme (RPBS) on subsidized medicines and estimates of non-subsidized medicines. RPBS data were calculated from continuous data on all prescriptions dispensed from a validated sample of community-based pharmacies. In-patient hospital prescribing is not included. Usage rate calculated on the basis of medication use of 1,000 persons per day. DDD, defined daily dose.

Although some quinolones have been approved for use in companion animals (Table 2), they have not been approved for use in food-producing animals in Australia. The National Registration Authority for Agricultural and Veterinary Chemicals (now the Australian Pesticides and Veterinary Medicines Authority) sought advice on the administration of enrofloxacin to pigs in the mid-1990s. The Expert Advisory Group on Antimicrobial Resistance advised that quinolones were an antimicrobial drug class of high importance in humans and should never be licensed for food-producing animals because of the risk for drug resistance in enteric pathogens and their potential transmission to humans through the food chain. This was supported by similar international guidance published at the time (*13*); the application was subsequently withdrawn. A range of quinolones have been registered in Australia for dogs and cats, but typing studies in Australia have so far demonstrated that quinolone-resistant *E. coli* found in humans are generally different from those in companion animals (*14*).

**Table 2:** Quinolone antimicrobial drugs available for use in companion animals in Australia under the authority of the Pharmaceutical Benefits Scheme

Drug (date registered)	Indication
Difloxacin (2001)	Treatment of infections in dogs caused by difloxacin-sensitive organisms
Enrofloxacin (2004)	Treatment of urinary and respiratory tract infections, deep pyodermas, wounds, abscesses, and discharging sinuses in dogs caused by enrofloxacin-susceptible bacteria
Orbifloxacin (1999)	Treatment of diseases in dogs and cats caused by orbifloxacin-sensitive bacteria
Ibafloxacin (2007)	Treatment of urinary tract, respiratory tract, skin, and soft tissue infections in dogs and cats caused by ibafloxacin-sensitive bacteria

In other countries where fluoroquinolones are used in food production, they are also often added to the drinking water provided for many food production animals, including poultry. In many middle-income countries, particularly those in Asia and Latin America, many quinolone antimicrobial drugs are licensed for use in cattle and poultry, and exposure in these animals is probably frequent and widespread (*8*,*13*). In the United States, quinolones were widely used in poultry until Food and Drug Administration officials withdrew approval for this use in 2005 because of the unacceptable resistance risk (*15*). In Europe, quinolone use remains a small proportion of antimicrobial drugs used in food animals (*16*).

## Low Resistance Rates in Australia Compared with Other Countries

Data for bacterial resistance are available from longitudinal studies performed by the Australian Group on Antimicrobial Resistance (www.agargroup.org/), a network of 30 participating laboratories representing all states and mainland territories that periodically perform nationally representative studies of bacterial isolates from hospitalized and nonhospitalized patientsError! Hyperlink reference not valid.. Quinolone resistance rates in disease-causing isolates of *E. coli* have remained consistently low in Australia. Before 2006, studies included isolates from both community-acquired and hospital-acquired *E. coli* infections and showed a rise in the percentage of isolates from 1992 (0.4%) to 1998 (1.0%) to 2006 (4.9%) (*17*). Since then, surveillance of isolates from community-acquired infections has shown resistance in 89 (4.1%) of 2,155 strains tested in 2008 (*18*) and 108 (5.2%) of 2,092 strains tested in 2010 (Australian Group on Antimicrobial Resistance, unpub. data). This finding is in contrast to data published regarding the United States, where resistance in urinary isolates increased from 3% to 17% during 2000–2010 (*19*), and Europe, where resistance correlated with antimicrobial drug use was described in as high as 45% of isolates in 2008 (*20*) (Figure 2). Similar ecological correlations have been observed between the use of quinolones in pigs and poultry and quinolone resistance in E. coli from humans (*21*).

**Figure 2:** Quinolone use data for Europe from the European Surveillance of Antimicrobial Consumption initiative for antibiotic use in ambulatory care settings and European Antimicrobial Resistance Surveillance System. Use data for Australia from the Australian Group on Antibiotic Resistance (community isolates) and Drug Utilization Sub-Committee Drug Utilization Database (Commonwealth of Australia). Line represents logit-modeled relationship between resistance and usage, weighted by number of isolates tested. Usage rate calculated on the basis of medication use of 1,000 persons per day. DDD, defined daily dose; *E. coli*, *Escherichia coli*.

Quinolone resistance in locally acquired *Campylobacter* spp. is relatively uncommon in Australia compared with most other countries. Several case–control studies in Australia showed that the proportion of disease caused by resistant *Campylobacter* spp. was low (0% in 2003 and 2.6% in 2006) and mainly attributable to resistant strains in returned travelers (*22*). Quinolone resistance in isolates of *Campylobacter* spp., *Salmonella* spp., and *E. coli* from a variety of food-producing animals and products is rare in Australia (*23*,*24*). Notably, there is no importation of fresh meat into Australia. This finding is in contrast to the situation in other countries where bacterial resistance in humans, food-producing animals, and products emerged in the 1990s, coincident with the increase in quinolone use among humans and animals, and led to the US Food and Drug Administration’s 2005 withdrawal of approval of quinolones for use in poultry production (*15*). Resistance rates >80% in *Campylobacter* spp. have been reported in some countries, such as Thailand (*25*). Quinolone resistance in *Campylobacter* spp. emerged in the United Kingdom after the licensing of enrofloxacin for veterinary use (*13*). This finding is consistent with those of the rapid emergence of resistance in *Campylobacter* spp. in chickens treated with quinolone (*26*).

In North America, quinolone resistance in pneumococci increased after levofloxacin was added to respiratory infection guidelines and its use became widespread (*27*). Resistance remains relatively low (*28*), probably reflecting the minimal use of quinolone in children, among whom pneumococcal carriage is more common than among adults. However, trends in ciprofloxacin-resistant *S. pneumoniae* closely parallel increased quinolone use in Canada, demonstrated by an increase in resistance from 0.6% in 1997 to 7.3% in 2006, and higher rates in elderly persons (*29*). In a similar manner, in Spain, ciprofloxacin resistance rose from 0.6% during 1991–1992 to 3.0% during 1997–1998, and was associated with an increase in quinolone use (*30*). By comparison, results from surveys in 2005 and 2007 by the Australian Group on Antimicrobial Resistance demonstrated low levels of antimicrobial drug resistance in Australia, demonstrated by moxifloxacin resistance detected in 0.1% of 1,775 isolates in 2005 and 0.2% of 1,814 isolates tested in 2007 (*31*).

An exception to the generally low rates of resistance in Australia is quinolone resistance in gonococci, which threatens to erode the effectiveness of treatment programs. In 2009, the Australian Gonococcal Surveillance Programme reported that 43% of tested gonococci were resistant to ciprofloxacin; however, ≈33% of those infections were acquired overseas (*32*). Resistance rates remain low in the Northern Territory, where acquisition is almost all local.

## Conclusions

Causation is sometimes defined by the counterfactual, or in this case, asking, “what would have happened if restriction had not occurred?” This question cannot conclusively be answered by ecologic data, and other factors are also likely to contribute to the observed heterogeneity between and within countries (*33*). However, it is notable that Australia, among the few countries to have successfully limited the use of quinolones in humans and prohibited their use in food-producing animals by public policy, and moreover has a safe water supply and a lack of imported uncooked meats from countries with high levels of resistant bacteria, remains among countries where quinolone resistance is low among virulent pathogens, such as *E. coli*. The link between fluoroquinolone use and resistance is supported by in vitro studies (*34*), analogy with other evolutionary processes, experimental studies in humans and animals (*5*,*6*), and ecologic studies showing a temporal association and a dose-response relationship between fluoroquinolone use and resistance (*20*).

Regulatory controls are probably the most effective, albeit crude, tool available to restrict antimicrobial drug use. Australia has had a long tradition of prescriber education through the National Prescribing Service and Therapeutic Guidelines. However, the effectiveness of educational initiatives is not clear because of the lack of published formal evaluations. Bulletins and letters about serious adverse events associated with flucloxacillin were sent to general practitioners by the Therapeutic Goods Administration, but they had minimal impact on prescribing volumes. Only subsequent restrictions imposed by the Therapeutic Goods Administration and PBS resulted in a 30% reduction in flucloxacillin use and 50% reduction in reported adverse hepatic events during 1994–1995 (*35*). However, the effectiveness of restriction of public subsidies as a tool to restrict human antimicrobial drug use is likely to be eroded as the price of antimicrobial drugs decreases (*36*). This measure is also not useful in influencing use of antimicrobial drugs in hospitals. To date, the relatively high cost of fluoroquinolones, such as ciprofloxacin, relative to other subsidized antimicrobial drugs, has meant that quinolone use has been restricted in Australia.

Data support the assertion that quinolone use in the community is low and use in hospitals in Australia is moderate. The Drug Utilization Sub-Committee Drug Utilization Database is used to estimate total outpatient antimicrobial drug use. This estimate was obtained by combining Medicare Australia data for government-subsidized medicines (PBS and Repatriation Pharmaceutical Benefits Scheme) with an estimate of nonsubsidized medicines, which is calculated from prescriptions dispensed from a validated sample of community-based pharmacies (*37*). Total use is converted to a rate of defined daily doses per person by using internationally standardized methods (*38*).

Although data from the early 1990s showed that retail sales for all antimicrobial drugs in Australia was higher than in countries in Europe, recent data show that quinolone use in Australia has remained lower than in countries in Europe (*37*) (Figure 2). The National Antimicrobial Utilization Surveillance Project collects data on broad-spectrum antimicrobial drug use in 32 hospitals. Usage data is based on purchasing data and suggests that although overall hospital use of these drugs in Australia is higher than that in Denmark, the Netherlands, and Sweden, quinolone use in Australia is similar to or lower than use in hospitals in these countries (*39*).

Have the limitations on prescribing quinolone in Australia had deleterious effects on human and animal health? Quinolones can still be prescribed when they are necessary or the indicated preferred treatment. Difficulties could be theorized where appropriate empirical therapy is delayed. However, in most of circumstances, empirical regimens in national prescribing guidelines indicate use of β-lactam agents, aminoglycosides, or macrolides and recommend quinolones only when microbiological confirmation of etiology and susceptibility testing demonstrates they are appropriate. The few circumstances where quinolones would be preferred empiric therapy (severe community-acquired pneumonia in patients with immediate hypersensitivity to penicillin) are usually accounted for in local stewardship protocols for severely ill patients in hospitals.

Regarding controls for food-producing animals, although the potential for antimicrobial resistance remains a criterion for Australian Pesticides and Veterinary Medicines Authority registration, external advice from the Expert Advisory Group on Antimicrobial Resistance ceased in 2004. Since then, other valuable classes of antimicrobial drugs used in humans have been registered for animal use, including third- and fourth-generation cephalosporins. Furthermore, there is no requirement for postmarketing surveillance of resistance for newly registered antimicrobial drugs in humans or animals. In an attempt to reestablish a coordinated and comprehensive approach to antimicrobial drug resistance in Australia, we recently proposed the formation of a national antimicrobial drug resistance management body to implement national surveillance, coordinate education and stewardship programs, implement infection-prevention and control policies, support research, and advise regulatory authorities (*40*). Our proposal is in concert with other recent international calls for urgent action on antimicrobial drug resistance.

We believe that this single example of coordinated public policy points the way toward ensuring that drug prescribing is controlled and appropriate. Antimicrobial drugs are unique because their use leads toward their inevitable ineffectiveness as resistance develops. There are few new agents in development, so there is an imperative to preserve the effectiveness of the currently available antimicrobial drugs for as long as possible. We believe that these precious drugs should be regulated differently from all other drugs and that a single regulating body should be used to license and restrict their use to the most appropriate circumstances in both human and animal health and agriculture. The evidence that the control of quinolone prescribing in Australia has led to the continued usefulness of this class of drugs as valuable antimicrobial agents is compelling, and it serves as a call for countries to review the way that antimicrobial agents are regulated and subsidized so that we can continue to treat infections in the future.

## References

[1] Acar JF, Goldstein FW. Trends in bacterial resistance to fluoroquinolones. Clin Infect Dis. 1997;24(Suppl 1):S67–73. 10.1093/clinids/24.Supplement_1.S678994781

[2] Ruiz J. Mechanisms of resistance to quinolones: target alterations, decreased accumulation and DNA gyrase protection. J Antimicrob Chemother. 2003;51:1109–17. 10.1093/jac/dkg22212697644

[3] Kaatz GW, Seo SM. Inducible NorA-mediated multidrug resistance in *Staphylococcus aureus.* Antimicrob Agents Chemother. 1995;39:2650–5. 10.1128/AAC.39.12.26508592996PMC163006

[4] Robicsek A, Jacoby GA, Hooper DC. The worldwide emergence of plasmid-mediated quinolone resistance. Lancet Infect Dis. 2006;6:629–40. 10.1016/S1473-3099(06)70599-017008172

[5] Horcajada JP, Vila J, Moreno-Martinez A, Ruiz J, Martinez JA, Sanchez M, Molecular epidemiology and evolution of resistance to quinolones in *Escherichia coli* after prolonged administration of ciprofloxacin in patients with prostatitis. J Antimicrob Chemother. 2002;49:55–9. 10.1093/jac/49.1.5511751767

[6] Gupta K, Hooton TM, Stamm WE. Isolation of fluoroquinolone-resistant rectal *Escherichia coli* after treatment of acute uncomplicated cystitis. J Antimicrob Chemother. 2005;56:243–6. 10.1093/jac/dki16915911548

[7] Apisarnthanarak A, Tunpornchai J, Tanawitt K, Mundy LM. Nonjudicious dispensing of antibiotics by drug stores in Pratumthani, Thailand. Infect Control Hosp Epidemiol. 2008;29:572–5. 10.1086/58749618510468

[8] Ho PL, Chow KH, Lai EL, Lo WU, Yeung MK, Chan J, Extensive dissemination of CTX-M–producing *Escherichia coli* with multidrug resistance to 'critically important' antibiotics among food animals in Hong Kong, 2008–10. J Antimicrob Chemother. 2011;66:765–8. 10.1093/jac/dkq53921393133

[9] Johnson JR, Sannes MR, Croy C, Johnston B, Clabots C, Kuskowski MA, Antimicrobial drug-resistant *Escherichia coli* from humans and poultry products, Minnesota and Wisconsin, 2002–2004. Emerg Infect Dis. 2007;13:838–46. 10.3201/eid1306.06157617553221PMC2792839

[10] Moulds R, Christiansen K, Currie B, Ferguson J, Franks G, Gilbert G, Therapeutic guidelines: antibiotic. Version 14, 2010. Melbourne (Australia): Therapeutic Guidelines Ltd; 2010.

[11] Mandell LA, Wunderink RG, Anzueto A, Bartlett JG, Campbell GD, Dean NC, Infectious Diseases Society of America/American Thoracic Society consensus guidelines on the management of community-acquired pneumonia in adults. Clin Infect Dis. 2007;44(Suppl 2):S27–72. 10.1086/51115917278083PMC7107997

[12] Gupta K, Hooton TM, Naber KG, Wullt B, Colgan R, Miller LG, International clinical practice guidelines for the treatment of acute uncomplicated cystitis and pyelonephritis in women: a 2010 update by the Infectious Diseases Society of America and the European Society for Microbiology and Infectious Diseases. Clin Infect Dis. 2011;52:e103–20. 10.1093/cid/ciq25721292654

[13] World Health Organization. Use of quinolones in food animals and potential impact on human health. Report and proceedings of a WHO meeting, Geneva, Switzerland, 2–5 June, 1998. Geneva. Organization. 1998.

[14] Platell JL, Cobbold RN, Johnson JR, Trott DJ. Clonal group distribution of fluoroquinolone-resistant *Escherichia coli* among humans and companion animals in Australia. J Antimicrob Chemother. 2010;65:1936–8. 10.1093/jac/dkq23620570998

[15] Price LB, Lackey LG, Vailes R, Silbergeld E. The persistence of fluoroquinolone-resistant *Campylobacter* in poultry production. Environ Health Perspect. 2007;115:1035–9. 10.1289/ehp.1005017637919PMC1913601

[16] Kools SA, Moltmann JF, Knacker T. Estimating the use of veterinary medicines in the European Union. Regul Toxicol Pharmacol. 2008;50:59–65. 10.1016/j.yrtph.2007.06.00318006198

[17] Turnidge J, Gottlieb T, Mitchell D, Pearson JC, Bell JM. Gram negative survey: 2006 antimicrobial susceptibility report. Adelaide (Australia): Australian Group on Antimicrobial Resistance; 2007.

[18] Australian Group on Antimicrobial Resistance. Gram negative survey: 2008 antimicrobial susceptibility report. Adelaide (Australia): The Group; 2011.

[19] Sanchez GV, Master RN, Karlowsky JA, Bordon JM. In vitro antimicrobial resistance of urinary *Escherichia coli* isolates among US outpatients from 2000 to 2010. Antimicrob Agents Chemother. 2012;56:2181–3. 10.1128/AAC.06060-1122252813PMC3318377

[20] Durham LK, Ge M, Cuccia AJ, Quinn JP. Modeling antibiotic resistance to project future rates: quinolone resistance in *Escherichia coli.* Eur J Clin Microbiol Infect Dis. 2010;29:353–6. 10.1007/s10096-009-0862-x20119677

[21] Vieira AR, Collignon P, Aarestrup FM, McEwen SA, Hendriksen RS, Hald T, Association between antimicrobial resistance in *Escherichia coli* isolates from food animals and blood stream isolates from humans in Europe: an ecological study. Foodborne Pathog Dis. 2011;8:1295–301. 10.1089/fpd.2011.095021883007

[22] Unicomb LE, Ferguson J, Stafford RJ, Ashbolt R, Kirk MD, Becker NG, Low-level fluoroquinolone resistance among *Campylobacter jejuni* isolates in Australia. Clin Infect Dis. 2006;42:1368–74. 10.1086/50342616619147

[23] Department of Agriculture. Fisheries and Forestry. Pilot surveillance program for antimicrobial resistance in bacteria of animal origin. Canberra (Australia): The Department; 2007.

[24] Obeng AS, Rickard H, Ndi O, Sexton M, Barton M. Antibiotic resistance, phylogenetic grouping and virulence potential of *Escherichia coli* isolated from the faeces of intensively farmed and free range poultry. Vet Microbiol. 2012;154:305–15. 10.1016/j.vetmic.2011.07.01021856098

[25] Cody AJ, Clarke L, Bowler IC, Dingle KE. Ciprofloxacin-resistant campylobacteriosis in the UK. Lancet. 2010;376:1987. 10.1016/S0140-6736(10)62261-121146103

[26] McDermott PF, Bodeis SM, English LL, White DG, Walker RD, Zhao S, Ciprofloxacin resistance in *Campylobacter jejuni* evolves rapidly in chickens treated with fluoroquinolones. J Infect Dis. 2002;185:837–40. 10.1086/33919511920303

[27] Chen DK, McGeer A, de Azavedo JC, Low DE. the Canadian Bacterial Surveillance Network. Decreased susceptibility of *Streptococcus pneumoniae* to fluoroquinolones in Canada. N Engl J Med. 1999;341:233–9. 10.1056/NEJM19990722341040310413735

[28] Patel SN, Melano R, McGeer A, Green K, Low DE. Characterization of the quinolone resistant determining regions in clinical isolates of pneumococci collected in Canada. Ann Clin Microbiol Antimicrob. 2010;9:3. 10.1186/1476-0711-9-320082699PMC2823643

[29] Adam HJ, Hoban DJ, Gin AS, Zhanel GG. Association between fluoroquinolone usage and a dramatic rise in ciprofloxacin-resistant *Streptococcus pneumoniae* in Canada, 1997–2006. Int J Antimicrob Agents. 2009;34:82–5. 10.1016/j.ijantimicag.2009.02.00219342204

[30] Liñares J, de la Campa AG, Pallares R. Fluoroquinolone resistance in *Streptococcus pneumoniae* [letter]. N Engl J Med. 1999;341:1546–7. 10.1056/NEJM19991111341201310577109

[31] Gottlieb T, Collignon P, Robson JM, Pearson JC, Bell JM. Antimicrobial susceptibility report of *Streptococcus pneumoniae* isolates from the Australian Group on Antimicrobial Resistance: 2007 surveillance report. Adelaide (Australia): The Group; 2009.

[32] Australian Gonococcal Surveillance Programme. Annual report of the Australian Gonococcal Surveillance Programme, 2009. Commun Dis Intell. 2010;34:89–95.2069020810.33321/cdi.2010.34.12

[33] Boyd LB, Atmar RL, Randall GL, Hamill RJ, Steffen D, Zechiedrich L. Increased fluoroquinolone resistance with time in *Escherichia coli* from >17,000 patients at a large county hospital as a function of culture site, age, sex, and location. BMC Infect Dis. 2008;8:4. 10.1186/1471-2334-8-418197977PMC2258293

[34] Barry AL, Jones RN. Cross-resistance among cinoxacin, ciprofloxacin, DJ-6783, enoxacin, nalidixic acid, norfloxacin, and oxolinic acid after in vitro selection of resistant populations. Antimicrob Agents Chemother. 1984;25:775–7. 10.1128/AAC.25.6.7756234858PMC185641

[35] Turnidge J. Antibiotic use or misuse? Med J Aust. 1997;167:116–7.926926110.5694/j.1326-5377.1997.tb138805.x

[36] Jensen US, Muller A, Brandt CT, Frimodt-Møller N, Hammerum AM, Monnet DL. Effect of generics on price and consumption of ciprofloxacin in primary healthcare: the relationship to increasing resistance. J Antimicrob Chemother. 2010;65:1286–91. 10.1093/jac/dkq09320363806

[37] McManus P, Hammond ML, Whicker SD, Primrose JG, Mant A, Fairall SR. Antibiotic use in the Australian community, 1990–1995. Med J Aust. 1997;167:124–7.926926510.5694/j.1326-5377.1997.tb138809.x

[38] Wertheimer AI. The defined daily dose system (DDD) for drug utilization review. Hosp Pharm. 1986;21:233–4, 239–41, 258.10317694

[39] Communicable DCB. National Antimicrobial Utilisation Surveillance Program: Annual Report 2009–10. Adelaide (Australia): Government of South Australia; 2011.

[40] Gottlieb T, Nimmo GR. Antibiotic resistance is an emerging threat to public health: an urgent call to action at the Antimicrobial Resistance Summit 2011. Med J Aust. 2011;194:281–3.2142627910.5694/j.1326-5377.2011.tb02973.x

# Review article: Control of Fluoroquinolone Resistance through Successful Regulation, Australia

**DOI:** 10.3201/eid1809.111515

**Authors:** Allen C. Cheng, John Turnidge, Peter Collignon, David Looke, Mary Barton, Thomas Gottlieb

**Affiliations:** Monash University, Melbourne, Victoria, Australia (A.C. Cheng);; Alfred Hospital, Melbourne (A.C. Cheng);; Women’s and Children’s Hospital, Adelaide, South Australia, Australia (J. Turnidge);; University of Adelaide, Adelaide (J. Turnidge);; The Canberra Hospital, Garran, Canberra, Australia (P. Collignon);; Australian National University, Canberra (P. Collignon);; Princess Alexandra Hospital, Brisbane, Queensland, Australia (D. Looke);; University of Queensland, Brisbane (D. Looke);; University of South Australia, Adelaide (M. Barton);; Concord Hospital, Sydney, New South Wales, Australia (T. Gottlieb);; and University of Sydney, Sydney (T. Gottlieb)

## Medscape CME Quiz

## Earning CME Credit

To obtain credit, you should first read the journal article. After reading the article, you should be able to answer the following, related, multiple-choice questions. To complete the questions (with a minimum 70% passing score) and earn continuing medical education (CME) credit, please go to www.medscape.org/journal/eid. Credit cannot be obtained for tests completed on paper, although you may use the worksheet below to keep a record of your answers. You must be a registered user on Medscape.org. If you are not registered on Medscape.org, please click on the New Users: Free Registration link on the left hand side of the website to register. Only one answer is correct for each question. Once you successfully answer all post-test questions you will be able to view and/or print your certificate. For questions regarding the content of this activity, contact the accredited provider, CME@medscape.net. For technical assistance, contact CME@webmd.net. American Medical Association’s Physician’s Recognition Award (AMA PRA) credits are accepted in the US as evidence of participation in CME activities. For further information on this award, please refer to http://www.ama-assn.org/ama/pub/category/2922.html. The AMA has determined that physicians not licensed in the US who participate in this CME activity are eligible for *AMA PRA Category 1 Credits*™. Through agreements that the AMA has made with agencies in some countries, AMA PRA credit may be acceptable as evidence of participation in CME activities. If you are not licensed in the US, please complete the questions online, print the certificate and present it to your national medical association for review.

## Article Title: Control of Fluoroquinolone Resistance through Successful Regulation, Australia

## CME Questions

**1. You are a public health official advising a health management organization regarding use of fluoroquinolones. Based on the review by Dr. Cheng and colleagues, which of the following statements about restrictions in Australia regarding use of fluoroquinolones is most likely correct?**

A. Quinolones are permitted in steer but not in cows

B. Ciprofloxacin is listed as an option in lower urinary tract infection only in patients with high fever

C. Moxifloxacin is listed as an option for empiric management of most cases of outpatient community-acquired pneumonia

D. Australia’s subsidized outpatient pharmaceutical scheme limits usage of relatively expensive drugs unless prescribed by doctors for indications listed in the Pharmaceutical Benefits Scheme

**2. Based on the review by Dr. Cheng and colleagues, which of the following statements about development of fluoroquinolone resistance in Australia compared to that in other countries is most likely correct?**

A. In *Echerichia coli*, quinolone resistance rates in disease-causing isolates have plateaued at a moderate level in Australia

B. Quinolone resistance in locally acquired *Campylobacter* spp. is about the same in Australia as in most other countries

C. An increase in quinolone resistance in pneumococci in North America was noted following the introduction of levofloxacin into respiratory infection guidelines and widespread usage

D. Quinolone resistance in isolates of *Campylobacter* spp., *Salmonella* spp., and *E. coli* from a variety of food-producing animals and products is not uncommon in Australia due to importation of meat

**3. Based on the review by Dr. Cheng and colleagues, which of the following statements about potential harms of restricting fluoroquinolone use, and strategies to eliminate those harms, would most likely be correct?**

A. Quinolones can no longer be prescribed at all in Australia

B. Most recommended empirical regimens use quinolones, and appropriate empirical therapy would therefore be delayed

C. No strategies are currently available for quinolone therapy in severely ill, hospitalized patients

D. Quinolones can still be prescribed in Australia when they are necessary or the indicated preferred treatment

## Activity Evaluation

**Table:** 

**1. The activity supported the learning objectives.**				
Strongly Disagree				Strongly Agree
1	2	3	4	5
**2. The material was organized clearly for learning to occur.**				
Strongly Disagree				Strongly Agree
1	2	3	4	5
**3. The content learned from this activity will impact my practice.**				
Strongly Disagree				Strongly Agree
1	2	3	4	5
**4. The activity was presented objectively and free of commercial bias.**				
Strongly Disagree				Strongly Agree
1	2	3	4	5
